# IRF7 Protects Against Severe Influenza A Infection Independently of TLR7 Recognition in Mice

**DOI:** 10.3390/v18091042

**Published:** 2026-09-20

**Authors:** Ana Karina Nisperuza Vidal, Chenxiao Wang, Michaela J. Allen, Xiaofeng Ding, Jefferson Fernandes Evangelista, Yilin Chen, Mst Shamima Khatun, Micaela R. Boxer Wachler, Calder R. Ellsworth, Mohammad Islamuddin, Robert Blair, Jay K. Kolls, Derek A. Pociask, Xuebin Qin

**Affiliations:** 1 Division of Comparative Pathology, Tulane National Biomedical Research Center, Covington, LA 70433, USA; anisperuzavidal@tulane.edu (A.K.N.V.); cwang27@tulane.edu (C.W.); xding4@tulane.edu (X.D.); ychen85@tulane.edu (Y.C.); cellsworth@tulane.edu (C.R.E.); mislamuddin@tulane.edu (M.I.); rblair3@tulane.edu (R.B.); 2 Department of Microbiology and Immunology, Tulane University School of Medicine, New Orleans, LA 70112, USA; jevangelista@tulane.edu (J.F.E.); mboxerwachler@tulane.edu (M.R.B.W.); 3 Department of Medicine and Pediatrics, Center for Translational Research in Infection and Inflammation, Tulane University School of Medicine, New Orleans, LA 70112, USA; mallen15@tulane.edu (M.J.A.); mkhatun@tulane.edu (M.S.K.); jkolls1@tulane.edu (J.K.K.); dpociask@tulane.edu (D.A.P.); 4 Department of Pulmonary Critical Care and Environmental Medicine, Tulane University School of Medicine, New Orleans, LA 70112, USA

**Keywords:** influenza A virus (IAV), Toll-like receptor 7 (TLR7), interferon regulatory factor 7 (IRF7), interferon response, antibody production

## Abstract

Influenza A virus (IAV) remains a major cause of respiratory morbidity and mortality worldwide. However, the precise role of the RNA sensor Toll-like receptor 7 (TLR7) and its downstream signaling mediator, interferon regulatory factor 7 (IRF7), during IAV infection remains elusive. To address this gap, we utilized single-cell RNA sequencing (scRNA-seq) alongside *Tlr7*-deficient and *Irf7*-deficient mouse models infected with the mouse-adapted IAV strain PR8. Pulmonary scRNA-seq of PR8-infected wild-type mice revealed robust upregulation of *Tlr7* and interferon (IFN) pathway-associated genes specifically in dendritic cells (DCs) and B cells, whereas *Irf7* induction occurred globally across all major lung compartments. Following PR8 infection, *Irf7*-deficient but not *Tlr7*-deficient mice exhibited significantly greater daily weight loss, increased mortality, and more severe bronchial epithelial hyperplasia compared to wild-type controls. Furthermore, infected *Irf7*-deficient mice displayed diminished early IFN-α and IFN-γ levels. Interestingly, both *Irf7*- and *Tlr7*-deficient strains demonstrated impaired anti-hemagglutinin (HA) antibody production. Notably, Tlr7 deficiency did not alter Irf7 protein expression or the production of IFNs and NF-κB in response to IAV, indicating that Tlr7 does not modulate Irf7 activation during infection. Collectively, these findings demonstrate that Irf7 protects against severe IAV pathogenesis independently of Tlr7 recognition in mice, whereas both Irf7 and Tlr7 are required for optimal anti-HA antibody production. **Importance**: Influenza A virus is a respiratory pathogen that remains a major threat to global health as a seasonal disease and a source of periodic pandemics. The outcomes of the infection can range from mild illness to severe pneumonia and death, particularly in vulnerable populations, yet the reasons why some individuals develop more severe disease are not fully understood. Early immune defenses in the lungs are critical for controlling the virus, but they can also contribute to harmful inflammation if not properly regulated. Key sensors that detect viral genetic material, such as TLR7, and the signaling pathways that activate antiviral responses, such as IRF7, play an essential role in shaping these outcomes. However, whether TLR7 is essential or redundant in IAV remains debated, and the independent role of IRF7 in IAV has not been directly demonstrated in mouse models. The significance of our study lies in defining how these early immune mechanisms influence the course of influenza A infection, providing insight that may guide the development of improved therapies for influenza and related respiratory viruses.

## 1. Introduction

The RNA viruses SARS-CoV-2 (CoV2) and influenza A (IAV) are respiratory viruses that continue to pose a global threat [1,2]. CoV2, a non-segmented, single-stranded, positive-sense RNA virus, infects host cells via its spike (S) protein binding to the angiotensin-converting enzyme 2 [3,4,5]. In contrast, IAV is a segmented, negative-sense, single-stranded RNA virus that infects host cells via its hemagglutinin (HA) protein, which binds to sialic acid receptors [3,6]. Although both viruses infect the respiratory epithelium, further investigation is needed to understand the similarities and differences in their pathogenesis and host immune responses, which will inform the development of more effective interventions for the diseases [3]. Toll-like receptors (TLRs) represent a class of evolutionarily conserved proteins pivotal in the innate immune system’s arsenal against pathogens, including CoV2 and IAV viruses [7,8]. By bridging innate and adaptive immunity, TLRs elicit a comprehensive immune response, ensuring a rapid and efficient reaction to microbial invasions [9]. Among the 10 TLRs in humans, the endosomal TLRs 3, 7, and 8 are critical to sensing RNA viruses [10]. TLR3 and TLR7 protein structures and functions are conserved in mice and humans [11,12,13]. They play crucial roles in recognizing viral nucleic acids and triggering innate immune responses. The activation of TLR3 and TLR7 signaling promotes phosphorylation and nuclear translocation of IRF7, resulting in robust interferon (IFN) production for antiviral defense [14,15,16,17,18].

Emerging clinical evidence indicates that the mutations of TLR7, encoded by an X-chromosome locus [19], contribute to the development of severe COVID-19 via reduced IFN response, mainly in plasmacytoid DCs [20,21,22,23,24]. The rare loss-of-function mutations of the *TLR7* gene attribute to approximately 1–2% of severe COVID-19 cases in males under the age of 60 without medical history [19,20,21,22,23,24]. Individuals carrying mutations of TLR7 downstream signaling molecule IRF7 [25] are also prone to viral infections of the respiratory tract [26]. Moreover, autoantibodies against type I IFN correlate with cellular immune alterations in severe COVID-19 in patients, which indicates that the reduced IFNs’ function may contribute to the severity of CoV2 infection [27]. Consistently, we documented that the deficiencies of either *Tlr7* or *Irf7* in mice increase the severity of COVID-19 through the reduced IFN production, resulting in a substantial reduction in the production of antibodies against CoV2 [28]. These findings highlight the importance of TLR7 and the downstream molecules such as IRF7 in innate and adaptive immunity in COVID-19.

In contrast, whether TLR7 is essential or redundant in IAV remains debated, and the independent role of IRF7 in IAV has not been directly demonstrated in mouse models. Previous studies emphasize the importance of TLR7 in enhancing innate immunity for antiviral defenses and promoting adaptive immunity for antibody production [29]. However, other research suggests that TLR7 is not critical in fighting IAV infection, with evidence showing that its absence has minimal impact on viral clearance or immune modulation [30]. A TLR7 antagonist restricts IFN-dependent immunopathology in a mouse model of severe IAV infection [31]. TLR7 promotes acute inflammation and lung dysfunction in mice infected with IAV but prevents late airway hyperresponsiveness [32]. These findings suggest that the role of TLR7 in innate immunity against IAV infection differs from its function in CoV2 infection, underscoring the need for further research. In addition, studies find that the activation of TLR7 and IRF7 promotes adaptive immunity for the induction of antibodies against heterologous strains of IAV and CoV2 [30,33]. The consensus is that TLR7 is dispensable for disease recovery in the acute stage of disease [30]. Nevertheless, the extent to which TLR7 and its downstream signaling contribute to the innate and adaptive immune responses to IAV remain poorly defined. To address this question, we mined our previously published scRNA-seq data obtained from the lungs of the mice either infected with or without the IAV PR8 (A/Puerto Rico/8/1934H1N1) infection [34] for exploring the transcriptional changes of TLRs and related signaling pathways at 4 and 6 days post-infection (DPI). We also utilized global *Tlr7*- and *Irf7*-deficient mice to explore the role of Tlr7 and Irf7 in the immune response to PR8 infection, a well characterized mouse-adapted strain of influenza A.

## 2. Results

### 2.1. IAV Infection Induces Upregulation of Tlr7 Transcripts

**Figure 1:**
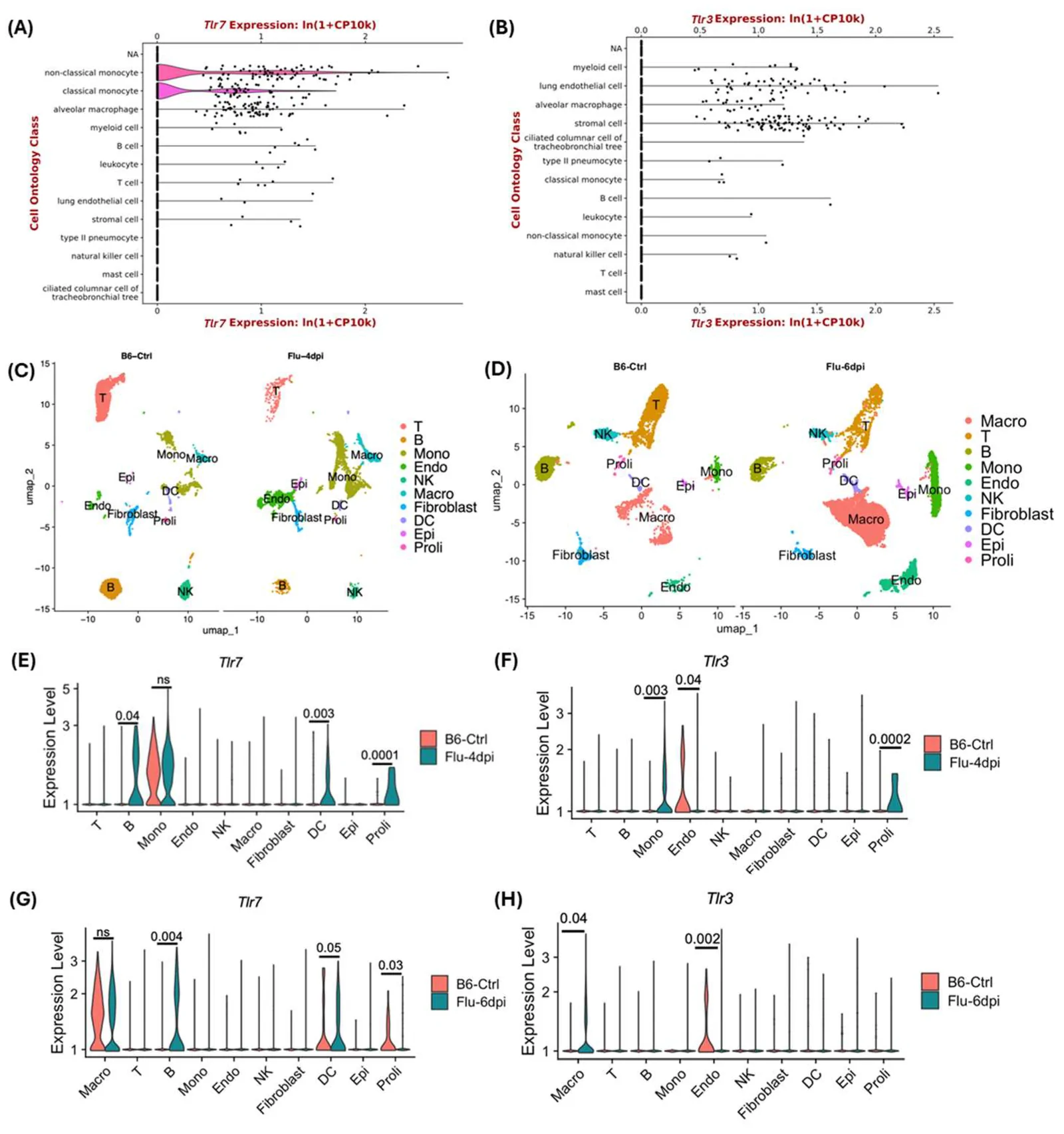
Upregulation of *Tlr7* during IAV infection. (**A**) Expression of *Tlr7* and (**B**) *Tlr3* across lung cell populations from scRNA−seq dataset (females, *n* = 4; males, *n* = 4) (The Tabula Muris Consortium, 2018). (**C**) Uniform manifold approximation and projection (UMAP) for dimension reduction plot with major cell types of scRNA−seq. Single−cell suspensions from whole infected lung from 12−week−old B6 non−infected controls (female, *n* = 2) and one 12−week−old IAV PR8-infected B6 mice (female, *n* = 1) at 4 DPI and (**D**) 6 DPI (female, *n* = 2). (**E**) Expression of *Tlr7* and (**F**) *Tlr3* across lung cell populations in controls and PR8-infected mice at 4 DPI. (**G**) Expression of *Tlr7* and (**H**) *Tlr3* across lung cell populations in control and PR8-infected mice at 6 DPI. Expression levels are shown as normalized single-cell expression values. Statistical significance between groups is indicated above each cell population; ns, not significant.

To define baseline expression patterns, we reanalyzed the Tabula Muris Consortium scRNA-seq dataset from lungs of 10–15-week-old C57BL/6JN (B6) mice [35]. *Tlr7* expression was mainly expressed in classical and non-classical monocyte subsets, as well as alveolar macrophages (Figure 1A and Supplementary Table S1A). In contrast, *Tlr3* expression was more broadly distributed across multiple cell types, including alveolar macrophages, myeloid cells, endothelial and stromal compartments (Figure 1B and Supplementary Table S1B). These results indicate a more cell-type-specific pattern for *Tlr7* versus a wider but sparse expression of *Tlr3* under steady state conditions.

To investigate the transcriptomic changes induced by IAV on *Tlr7*, we mined our previously published scRNA-seq data of mice lung tissue from uninfected and IAV-infected mice [34]. The infection was established using 50 PFU of IAV PR8 (A/Puerto Rico/8/1934H1N1) administered intranasally. *Tlr7* and *Tlr3* expressions were assessed at the single-cell level by analyzing scRNA-seq data of the lungs collected from PR8-infected mice at 4 and 6 days post-infection (DPI) [34]. Using uniform manifold approximation and projection (UMAP) for dimensionality reduction, we identified 10 distinct cellular clusters in both timepoints (Figure 1C,D). At 4 DPI, *Tlr7* was significantly upregulated in B cells and DCs (Figure 1E), whereas statistically significant differences in *Tlr3* expression were observed in monocytes (Figure 1F). This pattern of expression was consistent at 6 DPI, with a higher *Tlr7* expression in B and DC cells (Figure 1G) and a higher *Tlr3* expression in macrophages of PR8-infected lungs as compared to uninfected lungs (Figure 1H). Collectively, these findings demonstrate that there is a consistent upregulation of *Tlr7* in pulmonary immune cells during IAV infection.

### 2.2. Tlr7 Deficiency Does Not Affect Disease Severity in IAV Infection

**Figure 2:**
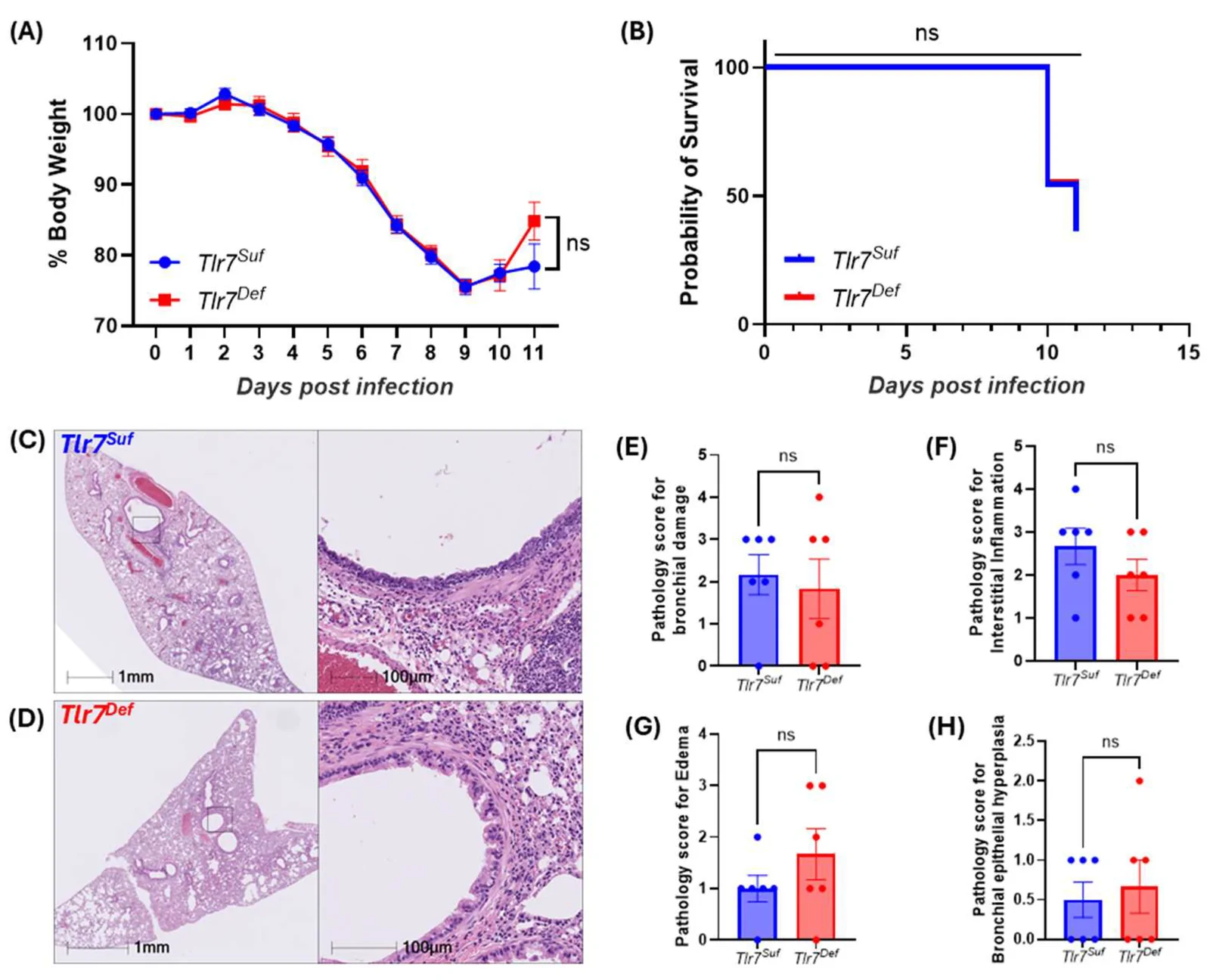
Global *Tlr7* deficiency does not affect mice recovery in IAV infection. (**A**) 12 to 16 weeks old *Tlr7^Suf^* mice (females, *Tlr7^+/+^*, *n* = 6; males, *Tlr7^+/Y^*, *n* = 5) and age−matched *Tlr7^Def^* mice (females, *Tlr7^−/−^*, *n* = 5; males, *Tlr7^−/Y^*, *n* = 4) were intranasally inoculated with IAV PR8 (83 PFU). Body weights were monitored daily and analyzed by a mixed-effects model (*p* = 0.2003). (**B**) Survival analysis of *Tlr7^Suf^* and *Tlr7^Def^* mice post PR8 infection. Survival curve was analyzed by the log-rank (Mantel−Cox) test (*p* = 0.7632). (**C**) Representative images of H&E staining in lungs of infected *Tlr7^Suf^* (females, *Tlr7^+/+^*, *n* = 3; males, *Tlr7^+/Y^*, *n* = 3) and (**D**) *Tlr7^Def^* mice (females, *Tlr7^−/−^*, *n* = 3; males, *Tlr7^−/Y^*, *n* = 3) at 9–11 DPI. (**E**) Pathology analysis did not reveal any differences for bronchial damage (*p* = 0.8701), (**F**) interstitial inflammation (*p* = 0.3723), (**G**) edema (*p* = 0.3961), or (**H**) bronchial epithelial hyperplasia (*p* > 0.999) between *Tlr7^Suf^* and *Tlr7^Def^* mice. Pathology comparisons were analyzed by unpaired *t*-test. Results are presented as mean ± SEM. ns, not significant.

To elucidate the role of Tlr7 in the development of severe IAV infection, we intranasally inoculated 12–16 weeks *Tlr7*-sufficient (*Tlr7^Suf^*) and *Tlr7*-deficient (*Tlr7^Def^*) mice in a B6 background with a lethal dose of PR8 (83 PFU). Because *Tlr7* is a X-linked gene, we refer to *Tlr7^+/+^* female and *Tlr7^+/Y^* male mice as *Tlr7^Suf^* and *Tlr7*^−/−^ female and *Tlr7*^−/Y^ male mice as *Tlr7^Def^*. Following infection, we tracked daily changes in body weight (Figure 2A) and assessed survival outcomes (Figure 2B). There were not any significant differences in the percentage of the daily weight loss and mortality rate in the infected *Tlr7^Def^* and *Tlr7^Suf^* mice (Figure 2A,B) with progressively decreasing body weight up to 9 DPI (Figure 2A) and 40% mortality rate at 11 DPI (Figure 2B). Histopathologic analysis did not reveal any significant differences regarding bronchial damage, interstitial inflammation, edema, or bronchial epithelial hyperplasia between the two groups (Figure 2C–H). In the lungs collected from the mice, we performed RT-qPCR analysis of matrix (M1) gene, a gold method for universal IAV detection and quantification [36], and documented that there was not any IAV viral load difference between the two groups (Supplementary Figure S1). Together, these findings demonstrate that *Tlr7* deficiency does not affect disease severity in IAV infection, indicating Tlr7 is dispensable for IAV infection recovery.

### 2.3. Irf7 Expression During IAV Infection

**Figure 3:**
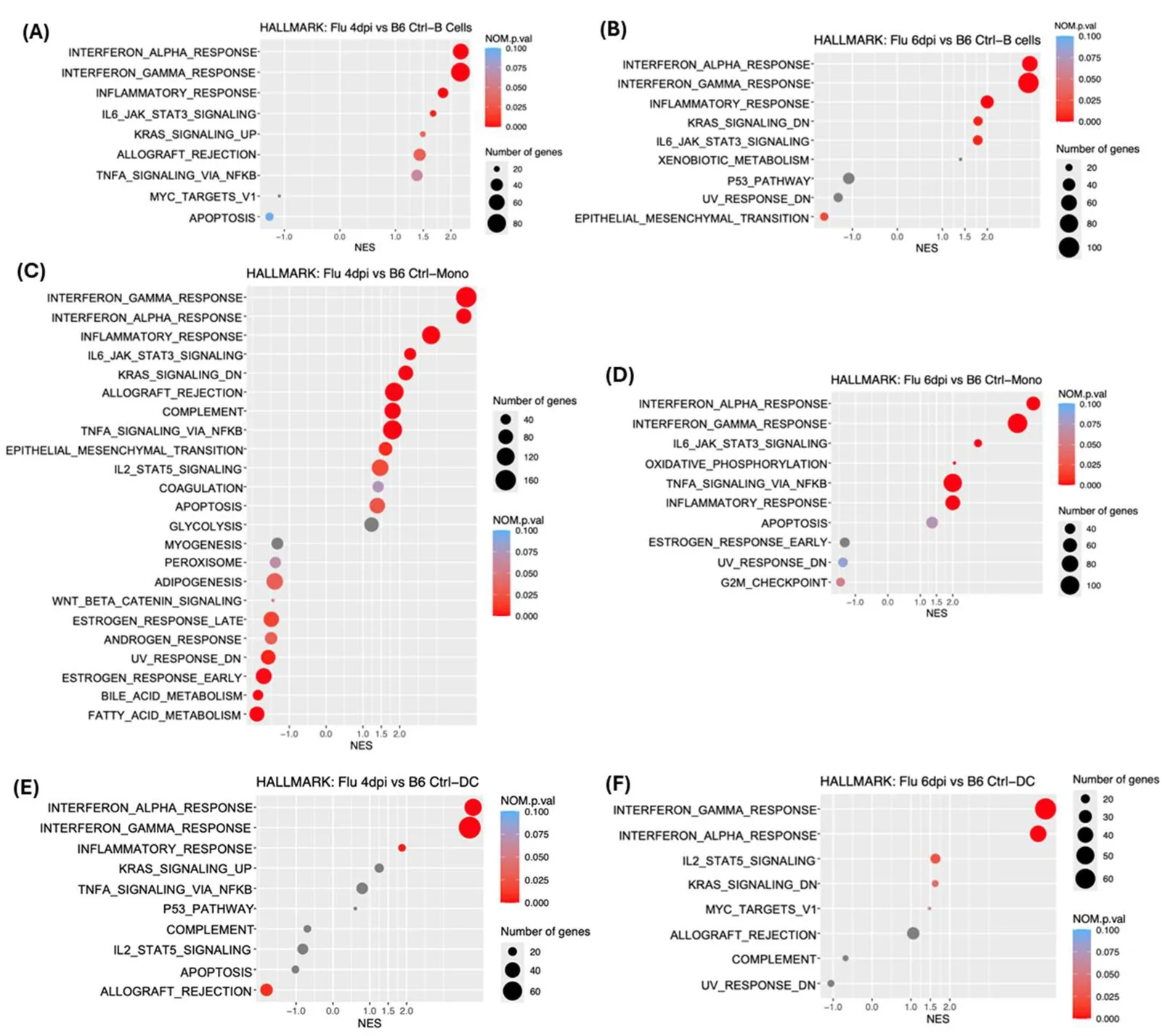
Upregulation of IFN responses in IAV infected mice. (**A**) Gene set enrichment analysis (GSEA) of Hallmark pathways in B cells comparing control B6 mice (female, *n* = 2) with IAV PR8 infected at 4 DPI (female, *n* = 1) and (**B**) 6 DPI (female, *n* = 2). (**C**) GSEA in monocytes at 4 DPI and (**D**) 6 DPI. (**E**) GSEA in dendritic cells at 4 DPI and (**F**) 6 DPI. Enrichment scores (NES) are shown on the x-axis. Dot size represents the number of genes contributing to each pathway, and color indicates normalized *p*-value (NOM p.val).

The Gene Set Enrichment Analysis (GSEA) of PR8-infected mice showed upregulation of genes related to the IFN gamma (IFN-γ) and IFN alpha (IFN-α) responses at 4 and 6 DPI in B cells (Figure 3A,B) and monocytes (Figure 3C,D) and DCs (Figure 3E,F). Additionally, GSEA showed IFN-γ and IFN-α responses upregulated in macrophages (Supplementary Figure S2A,B), epithelial (Supplementary Figure S2C,D) and endothelial cells (Supplementary Figure S2E,F). This GSEA results, together with our previous finding that TLR7-IRF7 axis plays a critical role in protecting against CoV2 infection [28], prompted us to explore the *Irf7* expression changes before and after IAV infection by mining our single-cell data [34].

**Figure 4:**
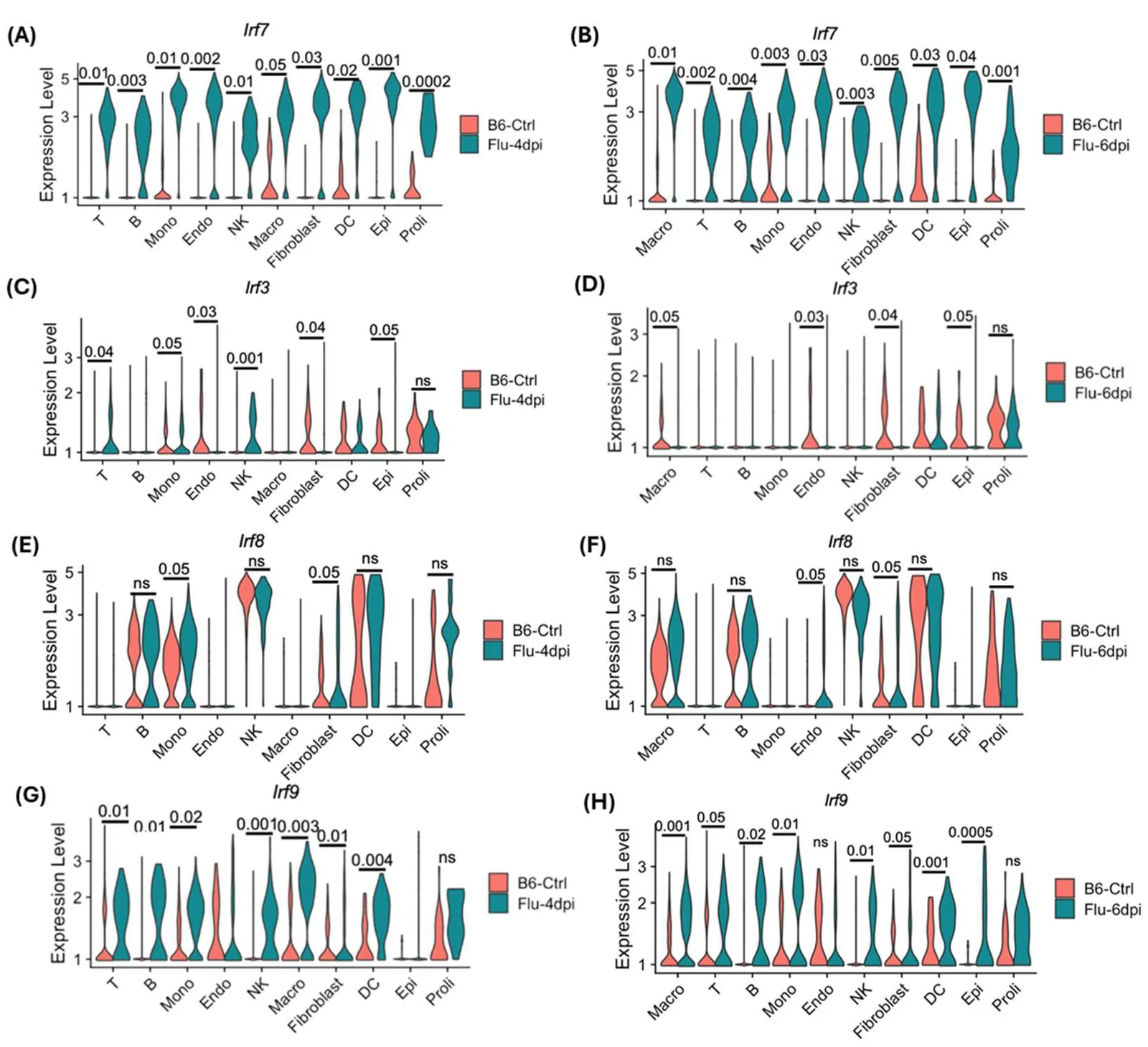
Expression of different *Irf* during IAV infection. (**A**) scRNA−seq analysis of *Irf7* expression across indicated lung cell populations in B6 controls (female, *n* = 2) and IAV PR8−infected mice at 4 DPI (female, *n *= 1) and (**B**) 6 DPI (female, *n* = 2), (**C**) *Irf3* at 4 DPI and (**D**) 6 DPI, (**E**) *Irf8* at 4 DPI and (**F**) 6 DPI, and (**G**) *Irf9* at 4 DPI and (**H**) 6 DPI. Expression levels are shown as normalized single cell expression values. Statistical significance between groups is indicated above each cell population; ns, not significant.

*Irf7* expression dramatically increased in the IAV-infected group both at 4 and 6 DPI in monocytes, macrophages, DCs, B cells, T cells, NK, epithelial, endothelial cells and fibroblasts (Figure 4A,B). Among the IRF family members, *Irf7* showed the broadest induction across cell populations. In contrast, *Irf3, Irf8*, and *Irf9* were upregulated in a more restricted subset of cell populations (Figure 4C–H).

The increased Irf7 expression was further confirmed at the protein level by Western blot, showing that IAV-infected B6 mice had significantly higher expression of Irf7 than non-infected B6 mice (Supplementary Figure S3A,B). These findings indicate that *Irf7* exhibits the broadest induction among IRF family members during IAV infection, consistent with its central role in orchestrating type I and type II IFN responses.

### 2.4. Irf7 Protects Against Severe Influenza A Infection

**Figure 5:**
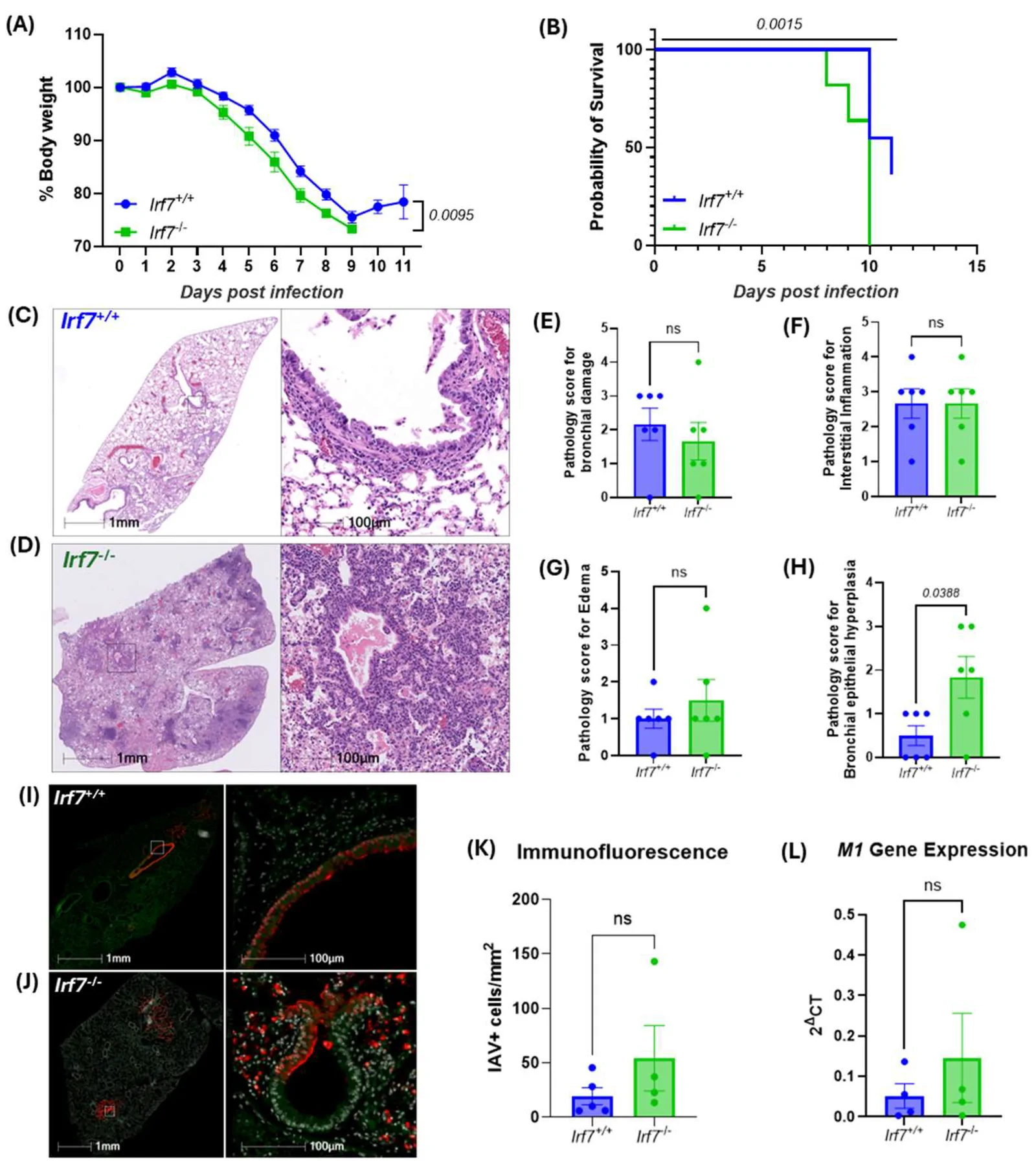
Global *Irf7* deficiency increases the severity of IAV infection in mice. (**A**) 12 to 16 weeks old *Irf7^+/+^* (female *n* = 6, male *n* = 5) and age-matched *Irf7^−/−^* (female *n* = 7, male *n* = 4) male mice were intranasally inoculated with IAV PR8 infection (83 PFU). Body weights were monitored daily and analyzed by a mixed-effects model (*p* = 0.0095). (**B**) Survival analysis by the Log-rank (Mantel-Cox) test (*p* = 0.0015). (**C**) Representative images and quantitative histology analysis of H&E staining of infected *Irf7^+/+^* (female *n* = 3, male *n* = 3) and (**D**) *Irf7^−/−^* mice (female *n* = 3, male *n* = 3) at 7–11 DPI. (**E**) Pathology analysis for bronchial damage (*p* = 0.3939), (**F**) interstitial inflammation (*p* > 0.999), (**G**) edema (*p* = 0.7576), and (**H**) bronchial epithelia hyperplasia *(p* = 0.0388). (**I**) Representative immunofluorescence images of lung sections from *Irf7^+/+^* (male, *n* = 5) and (**J**) *Irf7^−/−^* (male, *n* = 4) mice showing IAV staining (red) at 2 DPI. (**K**) Quantification of IAV-positive cells in lung sections by immunofluorescence, expressed as cells/mm^2^ (*p* = 0.2857). (**L**) Quantification of influenza A M1 gene expression in lung homogenates of infected *Irf7^+/+^* (male, *n* = 4) and *Irf7^−/−^* mice (male, *n* = 4) at 2 DPI by RT−qPCR, shown as 2^ΔCt^ (*p* = 0.6857). Scale bars, 1 mm (left) and 100 µm (right). Pathology and viral load experiments were analyzed by unpaired *t*-test and are presented as mean ± SEM. ns, not significant.

We further investigated the role of Irf7 in severe IAV infection by infecting 12–16-week-old *Irf7*-sufficient (*Irf7^+/+^*) and *Irf7*-deficient mice (*Irf7*^−/−^) in a B6 background with a lethal dose of PR8 (83 PFU) (Figure 5A–L). The infected *Irf7*^−/−^ lost more body weight up to 9 DPI (Figure 5A) and showed a higher mortality rate (Figure 5B) associated with higher bronchial epithelial hyperplasia (Figure 5C,D,H) than the infected *Irf7*^+/+^ control mice. However, no differences were found when comparing other histopathologic changes such as bronchial damage, interstitial inflammation, and edema (Figure 5C–G). No significant differences in viral load were observed between controls and *Irf7*^−/−^ mice at 2 DPI, as assessed by immunofluorescence staining of IAV nucleoprotein (Figure 5I–K) and RT-qPCR analysis of M1 gene expression in the lungs (Figure 5L). These results document that Irf7 protects against severe IAV infection, affecting survival and epithelial responses without altering viral burden.

### 2.5. Effect of Tlr7 and Irf7 Deficiencies in Adaptative Immunity During IAV Infection

**Figure 6:**
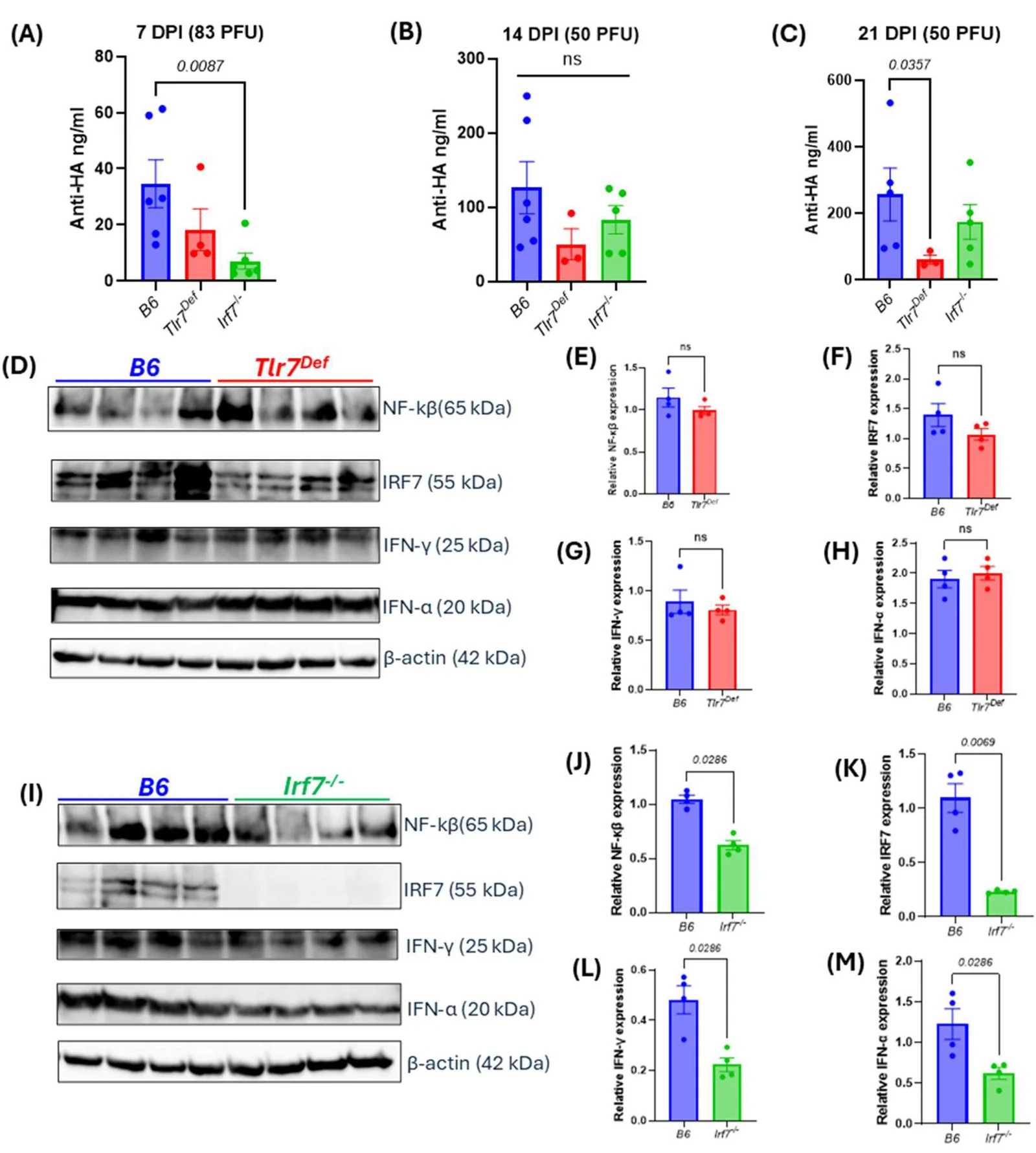
*Tlr7 and Irf7* deficiencies impair adaptive immunity against IAV. (**A**) Comparison of antibodies against hemagglutinin (Anti-HA, ng/mL) between B6, *Tlr7^Def^* and *Irf7^−/−^* mice at 7 DPI (83 PFU) (B6: male *n* = 3, female *n* = 3; *Tlr7^Def^*: male *n* = 2, female *n* = 2; and *Irf7^−/−^*: male *n* = 3, female *n* = 3). (**B**) Anti−HA levels at 14 DPI (50 PFU) (B6: male *n* = 3, female *n* = 3; *Tlr7^Def^*: male *n* = 1, female *n* = 2; and *Irf7^−/−^*: male *n* = 2, female *n* = 3). (**C**) Anti−HA levels at 21 DPI (50 PFU) (B6: male *n* = 3, female *n* = 3; *Tlr7^Def^*: male *n* = 1, female *n* = 2; and *Irf7^−/−^*: male *n* = 2, female *n* = 3). (**D**) Western blots of NF-κB (65 kDa), IRF7 (55 kDa), IFN-γ (25 kDa), and IFN-α (20 kDa) in lung lysates from B6 (male, *n* = 4 biological replicates) and *Tlr7^Def^* mice at 2 DPI (male, *n* = 4 biological replicates). β-actin (42 kDa) is shown as a loading control. (**E**) Densitometric quantification of NF-κB (*p* = 0.3429), (**F**) IRF7 (*p* = 0.3429), (**G**) IFN-γ(*p* = 0.8857), and (**H**) IFN-α(*p* = 0.8857) protein levels normalized to β-actin. (**I**) Western blots of NF-κB (65 kDa), IRF7 (55 kDa), IFN-γ (25 kDa), and IFN-α (20 kDa) in lung lysates from B6 (male, *n* = 4 biological replicates) and *Irf7^−/−^* (male, *n* = 4 biological replicates) mice at 2 DPI. β-actin (42 kDa) is shown as a loading control. (**J**) Densitometric quantification of NF-κB (*p* = 0.0286), (**K**) IRF7 (*p* = 0.0069), (**L**) IFN-γ (*p* = 0.0286), and (**M**) IFN-α (*p* = 0.0286) protein levels normalized to β-actin. Data are presented as mean ± SEM. Statistical significance was determined by unpaired *t*-test. ns, not significant.

Knowing that Tlr7 is dispensable for IAV infection recovery and Irf7 offers a protective effect against it, we further evaluated their effect in the adaptive immune response following IAV infection. IgG against the HA protein of IAV was measured by ELISA in mice infected with 83 PFU of PR8 to evaluate the acute phase of severe IAV infection. To measure antibody response in the subchronic and chronic phases of the IAV infection, we had to use non-lethal dose of PR8 (50 PFU) for the infection to assess antibody levels at days 14 and 21 [34,37]. We found that at 7 DPI after the higher dose, the infected *Irf7*^−/−^ showed a significantly lower level of anti-HA IgG than the infected control group, while *Tlr7^Def^* also tended to as well, but not at significantly lower levels than the controls (Figure 6A). Following infection with the sublethal dose, no differences were found among the B6 controls, *Tlr7^Def^*, and *Irf7*^−/−^ groups on day 14 post-infection (Figure 6B). At 21 DPI, *Tlr7^Def^* but not *Irf7*^−/−^ mice showed lower levels of anti-HA IgG than the control mice (Figure 6C). Of note, at all three points, both deficiencies showed a consistent trend toward lower antibody levels compared with their respective controls. These findings showed that both *Irf7*- and *Tlr7*-deficient mice had reduced anti-HA antibody production, which indicates that Irf7 and Tlr7 contribute to the development of adaptive immune responses to IAV infection.

### 2.6. IFN Production Is Reduced in Irf7^−/−^ but Not in Tlr7^Def^ Mice During Early IAV Infection

We further assessed how *Tlr7* and *Irf7* deficiencies affect the expression of Irf7, IFN-α, IFN-γ, and nuclear factor kappa-light-chain-enhancer of activated B cells (NF-κB) at the protein level during IAV infection by Western blot at 2 DPI (Figure 6D–M). NF-κB is another downstream signaling event of TLRs, such as TLR7 [38]. The infected *Tlr7^Def^* had similar protein levels of Irf7, IFN-α, IFN-γ, and NF-κB as the infected *Tlr7^Suf^* mice (Figure 6D–H). In contrast, the infected *Irf7^−/−^* mice had significantly lower IFN-α, IFN-γ, and NF-κB levels than the infected *Irf7^+/+^* mice (Figure 6I,J,L,M). As expected, we did not detect any Irf7 protein expression in the lungs of the infected *Irf7^−/−^* mice at 2 DPI (Figure 6K) and 7 DPI (Supplementary Figure S3A,B), further confirming the deficiency in Irf7 protein expression. These results demonstrate that Irf7 deficiency results in reduced IFN production, a downstream signaling event of Irf7 during early IAV infection, while *Tlr7* deficiency has no impact on Irf7 protein expression and downstream signaling events, such as NF-κB activation and IFN production at an early stage of infection. Additionally, we have also found that the infected *Tlr7^Def^* mice had a similar level of Irf7 induction as compared to the infected *Tlr7^suf^* mice in the lung collected at 7 DPI, a late phase of IAV infection (Supplementary Figure S3A,B). Taken together, these results support the notion that Irf7 protects against severe Influenza A infection independently of Tlr7 recognition in mice. Interestingly, we found that the deficiency of Irf7 caused reduced NF-κB protein during the early IAV infection, indicating that Irf7 may also modulate NF-κB besides IFN.

## 3. Discussion

Here, we report that the activation of Irf7 protects against severe IAV infection in mice by inducing early-phase IFN production. Supportively, the lethal PR8 dose of the infection triggered a higher mortality rate of the *Irf7^−/−^* mice (100% vs. 60%) with a greater weight loss as compared with the control mice. The bronchial epithelial hyperplasia observed in *Irf7^−/−^* mice may reflect a compensatory, yet pathological remodeling response to impaired IFN signaling—a phenomenon which warrants further investigation. After IAV infection, the *Irf7^−/−^* mice had lower IFN-α and IFN-γ levels than the control mice. Comparably, Wilk et al. also demonstrated that *Irf7* deletion renders the host highly susceptible to infection, leading to accelerated weight loss and reduced survival [39]. Similarly, Hatesuer et al. reported that *Irf7^−/−^* mice exhibit blunted type I IFN responses, increased weight loss, and diminished survival, highlighting its critical role in antiviral defense and immune regulation [40]. While a recent study conversely reported improved outcomes in *Irf7^−/−^* mice infected with a lethal dose of the PR8 strain [41], this discrepancy is likely attributable to differences in viral dosing and overall experimental design. Notably, human genetic evidence further underscores a central role for IRF7 in host defense; a patient with a near-fatal infection caused by the 2009 pandemic H1N1 virus was found to carry compound heterozygous, loss-of-function IRF7 mutations [42]. Additionally, an IAV-infected patient harboring a rare E331V missense variant in *IRF7* displayed compromised IRF7 activity, characterized by defective IFN priming and blunted antiviral responses in patient-derived cells [43]. Overall, these results, together with our findings reported here, establish IRF7 as a key component of host antiviral defense against IAV infection.

Using *Tlr7* and *Irf7* deficient mice at the same experimental setting, we further document that Tlr7 is dispensable for Irf7 activation and Irf7 downstream induction of Type I and II IFN immunity against IAV infection. Consistent with our findings, Jeisy-Scott et al. have also demonstrated that *Tlr7*-deficient mice are capable of mounting protective innate responses and controlling infection despite alterations in B cell responses [30]. We also observe that Tlr7 does not require sensing IAV for triggering the increased production of IFN-α, IFN-γ and NF-κB. These findings suggest that increased production during IAV infection may be attributed to the activation of alternative cytosolic RNA sensors in the airway, such as retinoic acid-inducible gene I (RIG-I) [29,44,45] and melanoma differentiation-associated protein 5 (MDA5) [46] for sensing IAV in airway. Supporting this hypothesis, both transcriptional (Supplementary Figure S4A,B) and translational levels (Supplementary Figure S4C) of RIG-I (also known as DEAD-box helicase 58, Ddx58) expression were elevated post IAV infection. This result indicates that RIG-I actively participates in the host response to IAV, corroborating previous reports identifying RIG-I as a critical RNA sensor for this virus [29,44,45]. Notably, RIG-I expression remained robust among infected B6, *Tlr7^Def^*, and *Irf7^−/−^* mice, demonstrating that its induction is independent of Tlr7 and Irf7 signaling (Supplementary Figure S4E–H). Previous studies have indicated that the sensing functions of RIG-I and MDA5 complement one another, with both sensors required to detect IAV infection in epithelial cells [46,47,48,49,50,51]. Taken together, our data suggest that IAV is primarily recognized by these cytosolic RNA sensors, culminating in rapid IRF7 activation and early IFN production to limit viral dissemination. Further investigation is required to delineate their discrete functional contributions, their synergistic interplay, and the localized activation patterns within specific airway regions necessary to mount an effective antiviral response against IAV.

Beyond innate immunity, we observed that the reduction in anti-HA antibody levels in both knockout models across all timepoints (7, 14, and 21 DPI) underscores the critical role of Tlr7 and Irf7 in mediating the adaptive immune response, as we observed in our COVID-19 model [28]. Whether these proteins act independently or synergistically to modulate antibody production requires further investigation. Nevertheless, several established, independent mechanisms could account for the impaired adaptive immunity observed in *Irf7^−/−^* mice. *Irf7^−/−^* mice exhibited a concurrent decrease in early inflammatory and antiviral components, as evidenced by the suppression of IFN-α, IFN-γ, and NF-κB at the early phase of IAV infection. IFN-α, IFN-γ, and NF-κB reduction could explain the reduced antibody production in *Irf7^−/−^* mice, since their activations are important for antibody production [28,38,52,53,54]. However, how IRF7 modulates these components in different cell compartments at the early phase of the infection to influence adaptive immunity needs to be further investigated. Regarding TLR7, this sensor is highly expressed in alveolar macrophages and DCs, with lower expression in the upper airway [55]. Because of this anatomical distribution, the activation of TLR7 during IAV infection is presumably delayed relative to other innate sensors, such as RIG-I and MDA5, that are highly expressed in the upper respiratory tract [56,57,58,59]. Previous studies have reported that Tlr7 activation provides intrinsic signaling required by immune cells for optimal antibody production [30,60]. How exactly TLR7 in alveolar macrophages and DCs detects IAV in the airway and participates in antibody production warrants further investigation.

While this study provides valuable insights, several limitations warrant consideration for future research. First, our current experimental design did not analyze viral restriction factors nor other RNA sensors such as MDA5 and TLR3, which could provide the additional innate immunity against the IAV infection. Furthermore, the use of global knockout mice precludes us to fully dissect the underlying cellular mechanisms, which requires further investigation with conditional knockout models. Finally, the scope of our conclusions is fundamentally defined by using a single Influenza A strain (PR8, H1N1); evaluating additional viral strains in future investigations will be critical to broaden our understanding of the pathogenesis of IAV infection.

## 4. Materials and Methods

### 4.1. Mice

*Irf7^−/−^* (RBRC01420) mice were purchased from RIKEN, Tsukuba, Japan. *Tlr7^Def^* (008380) and C57BL/6J (B6) were purchased from the Jackson Laboratory (Bar Harbor, ME, USA). All animals were housed in an animal facility at Tulane University (New Orleans, LA, USA).

### 4.2. Study Approval

All animal experiments were approved by the Institutional Animal Care and Use Committee at Tulane University under protocol 2288. We have complied with all relevant ethical regulations for animal use.

### 4.3. Influenza A Infection

Mice were infected with H1N1 A/PR/8/34 (PR8). Briefly, low (50 PFU) and high concentrations (83 PFU) were diluted in 50 uL of sterile saline, and mice were infected by oropharyngeal aspiration. Two infectious doses of PR8 were utilized to adequately assess both acute viral pathogenesis and long-term post-viral repair. The inoculum of 83 PFU was used to evaluate the acute phase (days 2–9), whereas a lower dose of 50 PFU was administered to reduce mortality and permit the evaluation of late-stage at days 14 and 21 [34,37]. Uninfected mice which served as the control group received sterile saline.

### 4.4. Tissue Collection and Process

The health status of the mice was monitored daily. Euthanasia was performed if the mice exhibit a body weight loss of 25% or upon reaching the predetermined necropsy date. Blood was drawn via cardiac puncture into a BD microtainer and allowed to clot at room temperature for a minimum of 30 min. Subsequently, the blood samples were centrifuged at 3000× *g* rpm for 10 min, and the serum was collected from the upper layer. One half of the lung was fixed in Z-FIX buffer at room temperature. The remaining lung tissue was divided as follows: half of the residual lung (left side) was immersed in 1 mL of Trizol reagent and stored at −80 °C for RNA extraction; the other half of the residual lung (left side) was frozen on dry ice without any medium and stored at −80 °C.

### 4.5. Histological Analysis and Quantification of Histopathologic Lesions

Fixed tissues were processed, cut, and stained with hematoxylin and eosin (H&E) and digitally scanned with Axio Scan.Z1 (Zeiss, Thornwood, NY, USA) by the Anatomic Pathology Core at Tulane National Biomedical Research Center. Tissue sections were assessed and scored by an independent pathologist who was strictly blinded to the experimental group allocations. Pathological damage was evaluated using a defined semi-quantitative scoring system ranging from 1 to 4 (+, ++, +++, ++++) based on the severity and extent of the observed lesions.

### 4.6. Immunohistochemistry

Tissue embedded in paraffin and fixed in formalin was sectioned at 4 μm, mounted on Superfrost Plus slides, and baked at 60 °C for 3 h. Sections were deparaffinized in xylene, rehydrated through graded ethanol, and rinsed in distilled water. Heat-induced epitope retrieval was performed by boiling slides for 20 min in Tris-based buffer (pH 9; Vector Labs H-3301, 0.1% Tween-20, Newark, CA, USA), followed by transfer to citrate buffer (pH 6.0; Vector Labs H-3300) and cooling to room temperature. Slides were washed in PBS and loaded onto a Ventana Discovery Ultra autostainer for blocking, primary antibody incubation (rabbit anti-FluA, GeneTex GTX125989, 1:1000, GeneTex Irvine, CA, USA), secondary antibody incubation, and Rhodamine-based detection, followed by DAPI counterstaining. Slides were washed, mounted with Mowiol/DABCO aqueous medium, and imaged at 20× using a Zeiss Axio Scan.Z1 scanner (Oberkochen, Germany).

### 4.7. Digital Image Analysis

Slides were stained with DAPI and Influenza A-specific marker, as indicated before, is imaged across three channels, including an empty channel to account for autofluorescence. Influenza A-labeled cells were quantified using the HighPlex FL v4.2.14 module in HALO v3.6. Regions of interest were drawn around the entire lung section. Thresholds for positive detection were set by a veterinary pathologist based on fluorescent intensity in each channel and subsequently checked for accuracy by the same pathologist.

### 4.8. RNA Isolation

Tissues were collected in 1 mL of Trizol reagent (Invitrogen, Cat. No. 15596026), and RNA was extracted using the RNeasy Mini Kit (QIAGEN, Cat. No. 74104, Venlo, The Netherlands) according to the manufacturer’s protocol. The RNA concentration was measured using the NanoDrop 2000 spectrophotometer (Thermo Fisher Scientific).

### 4.9. Subgenomic M1 Viral Detection

Viral burden was determined by RT-qPCR for viral matrix (M1) expression [36].

### 4.10. ELISA for Anti-HA Detection

At this stage, 96-well plates were coated overnight at 4 °C with recombinant HA protein (5 µg/mL; Influenza A/Puerto Rico/8/1934 H1N1 trimer, (Sino Biological Cat. No. 11684-V08H2, Beijing, China) in PBS. Plates were washed with PBST and blocked for 2 h at room temperature with 1% BSA and 0.1% Tween-20 in PBS. Serum samples and controls were added in duplicate at predetermined dilutions, along with a standard curve of Influenza A H1N1 HA Monoclonal Antibody (Invitrogen, Cat. No. MA5-29929, Carlsbad, CA, USA). After 1 h incubation and PBST washes, HRP-conjugated goat anti-mouse IgG (Southern Biotech, Cat. No 1036-05, Birmingham, AL, USA) was added for 30 min at 37 °C. Plates were washed, developed with TMB, stopped, and absorbance was read at 450 nm.

### 4.11. Western Blot

Lung tissues were harvested from mice at the indicated time points (2 or 7 days post-infection), snap-frozen, and homogenized in RIPA Lysis and Extraction Buffer (Thermo Fisher Cat. No 89900, Thermo Fisher Scientific, Waltham, MA, USA), containing protease and phosphatase inhibitors (Cell Signaling, Cat. No 5871S, Cell Signaling Technology, Danvers, MA, USA). Protein concentration was determined using a BCA assay Buffer (Thermo Fisher Cat. No 23227). Equal amounts of protein (60 µg per lane) were resolved on 4–20% (BioRad, Cat. No 5671094, Bio-Rad Laboratories, Hercules, CA, USA) and transferred onto PVDF membranes (BioRad, Cat. No 1620177). Membranes were blocked in EveryBlot Blocking Buffer (BioRad, Cat. No 12010020) for 10 min at room temperature and incubated overnight with primary antibodies against IRF7 (Cell Signaling, Cat. No 72073), NF-κB (Cell Signaling, Cat. No 6956), IFN-α (Thermo Fisher Cat. No PA5-119649), IFN-γ (ProteinTech, Cat. No 29788-1-AP, Rosemont, IL, USA), RIG-I (Cell Signaling, Cat. No 4520), and β-actin (Cell Signaling, Cat. No 3700) After washing, membranes were incubated with HRP-conjugated secondary antibody (Cell Signaling, Cat. No 7074) for 1 h at room temperature. Protein bands were visualized using SuperSignal™ West Atto Ultimate Sensitivity Substrate (Thermo Fisher Cat. No A38556) in a ChemiDoc™ Imaging System (BioRad, Cat. No 12003153). Protein levels were quantified by densitometry using Image J and normalized to β-actin as an internal loading control. All western blot experiments were performed using independent biological replicates (*n* = 4).

### 4.12. scRNA-Seq Tissue Processing and Library Preparation

Lungs were minced with forceps and small scissors and digested in 2 mL serum-free medium with 2 mg/mL collagenase (MilliporeSigma, MilliporeSigma, Burlington, MA, USA) and 80 U/mL DNase I (MilliporeSigma) for 60 min at 37 °C. A total of 5000 live cells per sample were targeted by using 10× Single Cell RNAseq technology provided by 10× Genomics (10× Genomics, Pleasanton, CA, USA). Full-length barcoded cDNAs were then generated and amplified by PCR to obtain sufficient mass for library construction. Pooled libraries at a final concentration of 1.8 pM were sequenced with paired-end single index configuration by Illumina NextSeq 550 (Illumina, San Diego, CA, USA).

### 4.13. scRNA-Seq Analysis

Raw FASTQ sequencing files were processed using Cell Ranger v7.1.0 to generate gene-expression count matrices. The resulting single-cell gene-expression matrices were subsequently analyzed using the Seurat package in R (Seurat suite version 2.2.1). Quality-controlled cells were normalized, and highly variable genes were identified for downstream analysis. Principal component analysis (PCA) was performed for dimensionality reduction, followed by Uniform Manifold Approximation and Projection (UMAP) for visualization of cellular populations. Cell types were initially annotated using Azimuth, a reference-based mapping approach, and cell-type assignments were further confirmed based on the expression of established canonical cell-type marker genes. Expression of selected genes was visualized across annotated cell populations using normalized expression values. Differential expressions between control, and IAV-infected cells were assessed using the Wilcoxon rank-sum test, as implemented in Seurat. Statistical significance was defined as *p* < 0.05.

### 4.14. Statistics and Reproducibility

The results presented represent data obtained from independent mice, with sample sizes indicated in the corresponding figure legends. For experiments in which subsets of available samples were analyzed, samples were selected using computer-based randomization. When samples from both sexes were available, selection was performed to ensure balanced representation of females and males across experimental groups. Infected mice were analyzed at multiple points post-infection to minimize bias and increase reproducibility. Data are presented as mean ± SEM. Comparisons among multiple groups over time were performed using two-way analysis of variance (ANOVA), followed by Bonferroni’s post hoc test. Comparisons between two groups were performed using Student’s *t*-test. Statistical significance was defined as *p* < 0.05.

## Data Availability

The datasets used during the current study are available from the first author or corresponding author. The scRNA-seq data reported in this paper have been deposited to the GEO database, accession number: GSE248778.

## Supplementary Materials

The following are available online at https://www.mdpi.com/article/10.3390/v18091042/s1, Table S1: Summary statistics of *Tlr7* and *Tlr3* expression across lung cell populations. Figure S1: M1 viral gene expression. Figure S2: GSEA. Figure S3: IRF7 expression in IAV infection. Figure S4: RIG-I expression in IAV infection.
