# Supplementary material for: IRF7 Protects Against Severe Influenza A Infection Independently of TLR7 Recognition in Mice
Source: Viruses. 2026 Sep 20;18(9):1042. doi: 10.3390/v18091042 (PMC13611839; doi:10.3390/v18091042)
Supplement: Supplementary file 1 [file viruses-18-01042-s001.zip › V_Supplementary_Figures.pdf]

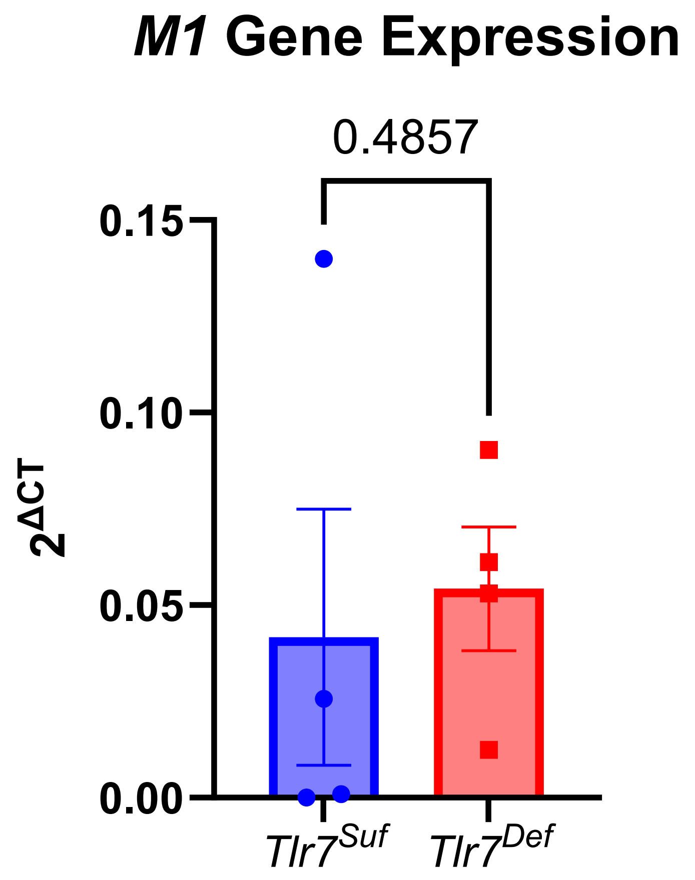

Supplementary Figure S1. M1 viral gene expression. Relative expression of influenza A virus matrix gene (*M1*) in lung tissue from *Tlr7*^{Suf} vs. *Tlr7*^{Def} mice. Viral RNA levels were quantified by qPCR and normalized using the $2^{\Delta Ct}$ method. Each point represents an individual mouse; bars indicate mean ± SEM.

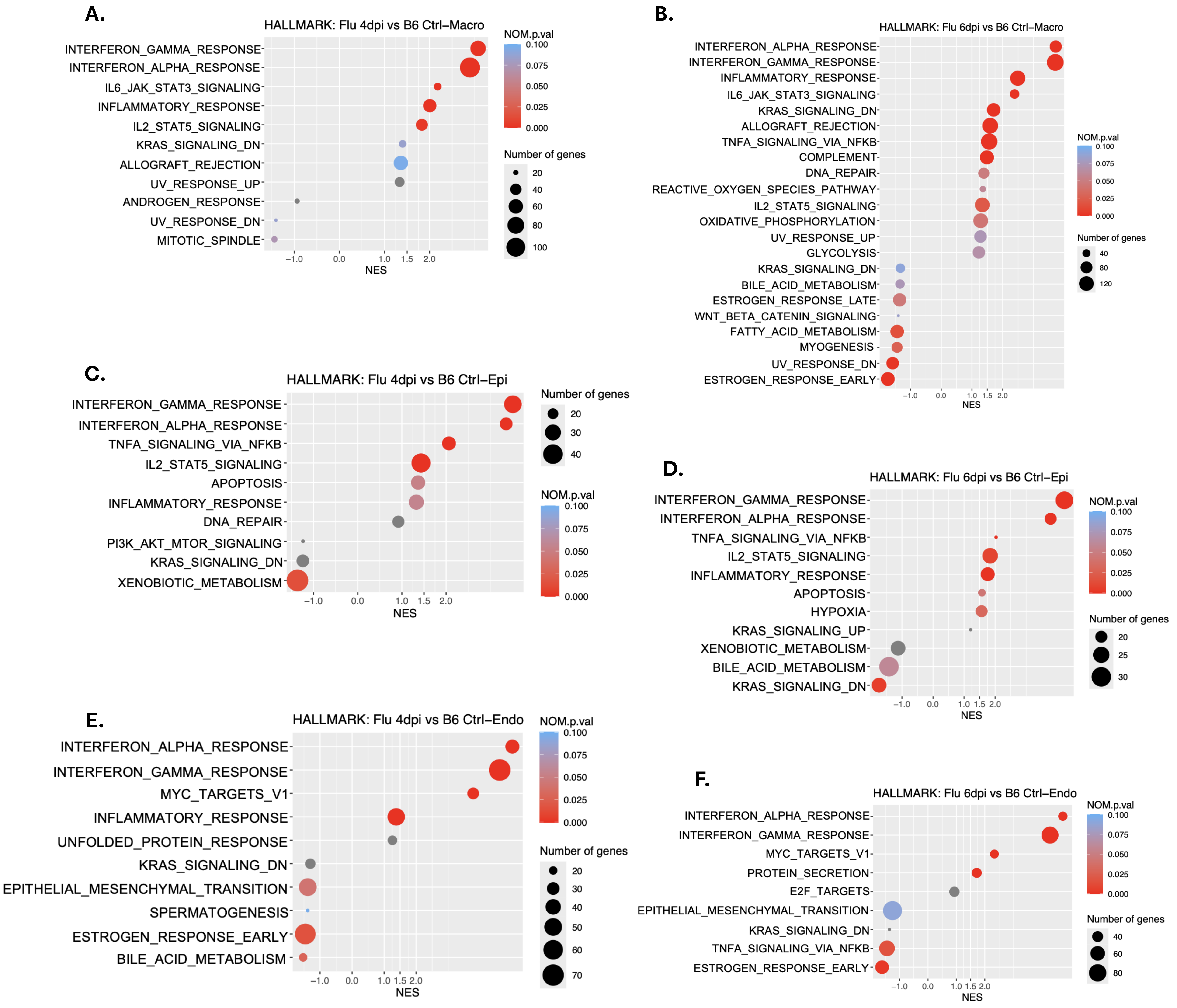

Supplementary Figure S2. GSEA. A–B) Gene set enrichment analysis (GSEA) of Hallmark pathways in macrophages from IAV infected versus B6 control mice at 4 dpi (A) and 6 dpi (B). **C–D)** GSEA of Hallmark pathways in epithelial cells comparing IAV infected and B6 control mice at 4 dpi (C) and 6 dpi (D). **E–F)** GSEA of Hallmark pathways in endothelial cells comparing IAV infected and B6 control mice at 4 dpi (E) and 6 dpi (F). Normalized enrichment scores (NES) are shown on the x-axis. Dot size represents the number of genes contributing to each pathway, and color indicates normalized *p*-value (NOM p.val)

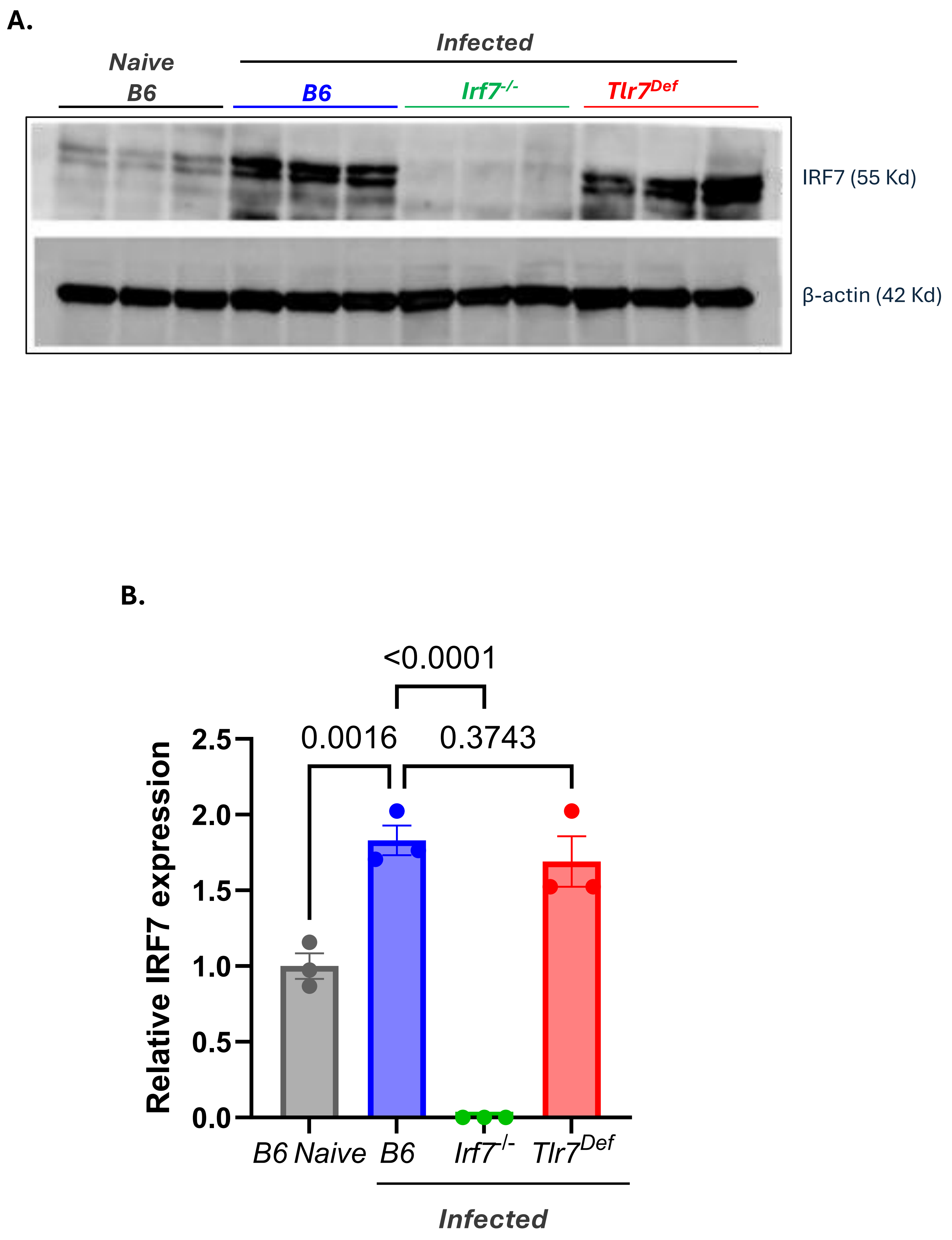

Supplementary Figure S3. IRF7 expression in IAV infection. A) Western blot of IRF7 (55 kDa) in lung lysates from naïve **B6**, and infected-*B6, Irf7*$^{-/-}$, and *Tlr7*^{Def} mice at 7 DPI. β-actin (42 kDa) is shown as a loading control. **B)** Densitometric quantification of IRF7 protein levels normalized to β-actin and expressed relative to naïve controls. Data are presented as mean ± SEM. Statistical significance was determined by unpaired *t*-test. *$p < 0.05$, ** $p < 0.01$, and *** $p < 0.001$, *ns,* not significant.

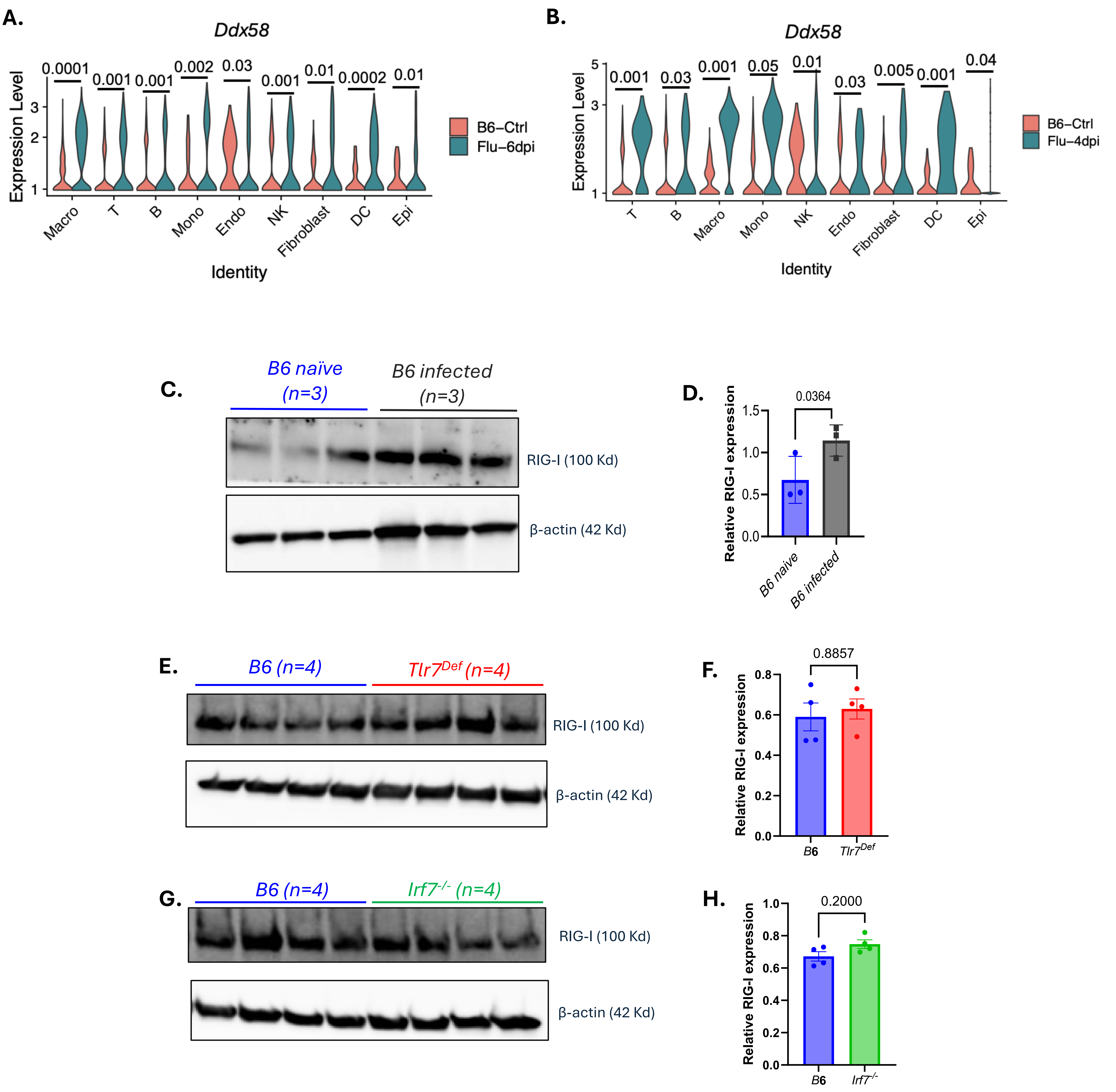

Supplementary Figure S4. RIG-I expression in IAV infection. A - B) Single-cell RNA-seq analysis showing Ddx58 (RIG-I) expression across indicated lung cell populations in B6 control and influenza A infected mice at 4 dpi (A) and 6 dpi (B). **C–H)** Western blots and corresponding densitometric quantification of RIG-I (100 kDa) in lung lysates from B6 naïve and B6 infected (C–D), B6 and *Tlr7^{Def}* (E–F), and B6 and *Irf7$^{-/-}$*(G–H) mice at 2 DPI. β-actin (42 kDa) is shown as a loading control. Protein levels were normalized to β-actin and expressed relative to B6 controls. Data are presented as mean ± SEM. Statistical significance was determined by unpaired two-tailed Student's *t*-test (Figures F, H) and one-tailed Student's *t*-test (Figure D); ns, *not significant*.
