# Supplementary material for: IRF7 Protects Against Severe Influenza A Infection Independently of TLR7 Recognition in Mice
Source: Viruses. 2026 Sep 20;18(9):1042. doi: 10.3390/v18091042 (PMC13611839; doi:10.3390/v18091042)
Supplement: Supplementary file 1 [file viruses-18-01042-s001.zip › V_Supplementary_Table.pdf]

A.

Tissue	Mean	Median	Median > 0	# cells	% 0	% > 0
non-classical monocyte	0.42	0	1.15	220	65.00	35.00
classical monocyte	0.27	0	0.77	161	68.94	31.06
alveolar macrophage	0.20	0	0.92	345	78.26	21.74
myeloid cell	0.06	0	0.79	87	91.95	8.05
B cell	0.04	0	1.34	204	97.06	2.94
leukocyte	0.03	0	1.09	152	97.37	2.63
T cell	0.03	0	1.00	247	97.57	2.43
lung endothelial cell	0.01	0	0.84	462	99.35	0.65
NA	0.00	0	NA	45	100.00	0.00
stromal cell	0.00	0	1.05	2540	99.84	0.16
type II pneumocyte	0.00	0	NA	89	100.00	0.00
natural killer cell	0.00	0	NA	824	100.00	0.00
mast cell	0.00	0	NA	24	100.00	0.00
ciliated columnar cell of tracheobronchial tree	0.00	0	NA	49	100.00	0.00

B.

Tissue	Mean	Median	Median > 0	# cells	% 0	% > 0
myeloid cell	0.12	0	1.10	87	87.36	12.64
lung endothelial cell	0.12	0	1.16	462	90.04	9.96
alveolar macrophage	0.06	0	0.86	345	92.75	7.25
stromal cell	0.04	0	1.19	2540	96.46	3.54
ciliated columnar cell of tracheobronchial tree	0.03	0	1.39	49	97.96	2.04
type II pneumocyte	0.03	0	0.68	89	96.63	3.37
classical monocyte	0.01	0	0.69	161	98.14	1.86
B cell	0.01	0	1.61	204	99.51	0.49
leukocyte	0.01	0	0.94	152	99.34	0.66
NA	0.00	0	NA	45	100.00	0.00
non-classical monocyte	0.00	0	1.06	220	99.55	0.45
natural killer cell	0.00	0	0.78	824	99.76	0.24
T cell	0.00	0	NA	247	100.00	0.00
mast cell	0.00	0	NA	24	100.00	0.00

Supplementary Table S1. Summary statistics of *Tlr7* and *Tlr3* expression across lung cell populations. (A) Quantitative summary of *Tlr7* expression across annotated lung cell populations from the droplet-based single-cell RNA-seq dataset of the Tabula Muris Consortium[31]. For each cell type, mean expression, median expression, median expression among expressing cells (>0), total number of cells analyzed, percentage of cells with zero expression (% 0), and percentage of cells with detectable expression (% >0) are shown. **(B)** Quantitative summary of *Tlr3* expression across the same lung cell populations. Metrics are defined as in (A).
